# Treatment of Abortion

**Published:** 1897-02

**Authors:** Q. C. Smith

**Affiliations:** Austin, Texas


For the Texas Medical Journal.

### TREATMENT OF ABORTION.

BY Q. C. SMITH, M. D., AUSTIN, TEXAS.

Discussion of a paper on the subject by Dr. W. A. Harper, read before the Travis County Medical Society November 5, 1896. Abstract of Proceedings.

DR. W. A. HARPER, of Austin, read a paper on abortion. Discussion opened by Dr. W. J. Mathews. He said: In cases where a vaginal tampon was necessary, it should be composed of candle-wicking, and saturated with carbolic acid. But as the greatest dangers from abortions, was septic poisoning, the uterus should be emptied promptly, and the entire endometrium thoroughly scraped with a sharp curette; then the endometrium and vagina thoroughly mopped with carbolic acid; then the uterine cavity and vagina well packed with iodoform gauze, and held firmly in place with T bandage.

This process of disinfection and packing to be repeated each and every day, as long as any perceptible febrile movement obtains.

That should a physician be called *early* in a case of abortion, before any febrile disturbance, or septic complications had occurred, and such physician failed or neglected to promptly carry out aforesaid line of curetting, disinfecting and packing treatment, and the patient should die, that said physician should be held guilty of manslaughter.

Dr. Q. C. Smith said: He thanked the author, Dr. Harper, for his excellent paper, which had followed on the lines of latest standard works, and manifested industry, earnestness and diligent research in its preparation.

Mr. President, I have never lost a case of abortion from hemorrhage or septic poisoning, if I had charge of the case from the beginning of the aborting disturbance. And the large majority of the few fatal cases of abortion that I have been called to treat, were cases in which the mother's parts had previously received more or less traumatic injury; so we should in *all* cases of abortion, very carefully avoid even the slightest trauma of the mother's parts. But to go over the whole subject of abortion, would consume too much time, for the present occasion. Each case of abortion should be regarded as a new problem, and the details of its treatment, applied to meet the indications and requirements of each given case.

And as the conditions and environments of cases of abortion, are often so diverse, it is impossible that *any* one routine and exact plan of their treatment, can be laid down upon hard and fast lines, as the *only* proper, and best treatment for *all* cases of abortion; or even for a large majority of such cases. But to be brief, yet specific as possible, we would suggest two typical cases of abortion and their treatment. One, a plain uncomplicated case; the other, complicated with septic conditions and ptomainic poisoning.

The plain case: One occurring in the early months of pregnancy, the patient's previous health good. From some non-traumatic cause, such patient begins to bleed from the uterus, more or less, the physician being soon called. Premising that it is desired to complete the abortion quickly and safely, we would, probably, proceed as follows: Give hypodermic of ½ grain morphia, 1-50 gr. atropia, and two grains quinine, immediately. Place patient in Sims' position, introduce Sims' speculum, very carefully dilate cervix with Sims' dilator, tampon the vagina closely with absorbent cotton. Immediately begin giving full doses of pleasant saline cathartics, dose every hour, until bowels move freely, using clysters, if necessary. Usually, the hypodermic eases pain, and quiets the patient for a time.

But as a rule, after a few hours, often sooner, the uterine contractions become stronger, more regular and effective; and if the tampon be well placed, it confines the blood in the uterine cavity, strongly tending to detach the placenta from the uterine wall; the uterine cavity (in such cases) being too small to contain a lethal amount of blood, there need be no fears of dangerous hemorrhage within the uterus. Hypodermics of quinine, 2 to 4 gr., should be repeated every 3 to 6 hours, until the uterus is emptied; then such constitutional treatment to follow as may be indicated in any given case.

After a few hours, or sooner, if indicated, remove the vaginal tampon—if nature has not already driven it, with uterine contents, out—and if uterine contents is not already expelled or easily removed, repeat vaginal tampon, packing very *closely*. But it is rare, if first vaginal tampon is well placed and closely packed, that it fails to cause the uterus to expell its contents entire, or within easy reach of complete romoval.

After all is removed, carefully mop out the vagina with absorbent cotton, put patient on fresh clean dry bed, and treat her much the same as in after-treatment of ordinary obstetric case.

Now for the complicated septic case of abortion. Of course it is well known that this class of cases occur in great variety of conditions and circumstances. Some cases in which grave septic manifestations supervene within forty-eight hours after first pronounced signs of abortion occur.

Again, patients have carried a more or less decomposed fœtus in utero more than a year, without alarming illness resulting; the fœtus, in some such prolonged cases, becoming more or less calcified, especially those surfaces most closely pressed against the uterine wall. I have seen two such partially calcified cases, in my own practice.

But to briefly outline an ordinary septic case of abortion, in which the fœtus and membranes, in more or less decomposed state, remain in uterus, and pronounced septic symptoms are manifest.

First, give hypodermic morphia ½ gr., strychnia 1-50 gr., and 2 gr. quinine; and 2 or 3 ounces of whisky in warm toddy. Place patient in Sims' position—preferably on high table, and before good light—introduce Sims' speculum, and very carefully mop the vagina clean. Very gently and carefully dilate cervix uteri—which is usually soft and easily dilatable in such cases—

then remove uterine contents with whatever instruments are best suited to the case; always using the gentlest means, and avoiding every liability of causing even the slightest traumatism to the mother's parts. After the solid and tougher parts of the uterine contents have been removed,—the skull often giving most trouble—then with dry absorbent cotton, mop out the entire uterine cavity and vagina clean and dry. Then immediately, very carefully but thoroughly, mop over the entire endometrium and vagina, with a mixture of Churchill's tincture of iodine, with one dram each, hydrate chloral and camphor, to each ounce.

We thus use this iodine compound because it is a powerful and harmless antiseptic, penetrates the tissues better than silver nitrate and other similar medicaments, as it so combines with the tissues, as not to be absorbable therefrom, hence, cannot be toxic, while it sears over the surfaces hitherto bathed in ptomainic toxines, thus effectually preventing their absorption into the circulation. Now place patient in clean, dry, fresh bed, and administer such remedies as may be indicated in the case; always remembering that quinine and carb. ammonia are powerful constitutional antiseptics, and strychnia hypodermically, the most potent drug to prevent or cure puerperal tetanus—a frequent, and often fatal complication of puerperal septic poisoning.

The bowels should be moved once or twice every twenty-four hours, preferably with salines—phosphate soda being a favorite with us.

Now, to reply briefly as possible, to some leading points set forth by Dr. Mathews, I beg to say:

1st. The *sharp* curette to scrape the endometrium: Any instrument likely to cause trauma, should not be used in the uterine cavity, in cases of abortion, but especially so in *septic* cases. As in such cases, even the slightest traumatism, may open the door of the absorbents and permit the egress of enough ptomainic poison to kill the patient.

2d. Carbolic acid as a surgical antiseptic: So many disastrous results and injurious effects come to pass from the use of carbolic acid as a surgical antiseptic, that its chief apostle (whom it had made *famous*, if not great), entirely abandoned its use many years ago, and so publically announced in medical journals and verbally, on many public occasion. And at latest, and very recent accounts, Sir Joseph was still anxiously searching for a substitute for carbolic acid—something that would prevent or kill microbes, yet be at least, harmless to the patient. And for years before the late Prof, Keith died, he totally and publicly abandoned and condemned the use of carbolic acid as a surgical antiseptic, and after its total abandonment, and without the use of *any* other surgical antiseptic, he secured even better results, than he had ever obtained whilst using carbolic acid, or any other surgical antiseptic. And it is well known that for years before Surgeon Keith abandoned carbolic acid, it so injured his own health, that he was compelled to leave off work .every few months to rest and recuperate. And he repeatedly publicly stated, that he felt sure that his health had been permanently injured, and his life shortened, by carbolic acid poisoning—from using it on his patients—and that it had injured many of his patients, and caused the death of a few of them. So, this great and noble man, and eminently capable and successful suigeon, lived to learn of, and publicly acknowledge the direful mistakes his enslavement to a *theory* had led him to make, and bitterly repented the day he placed carbolic acid in his surgical armamentarium. And many years ago the large wool-growers of California abandoned carbolic acid as a sheep-wash for scab, and other ovine cutaneous diseases, because carbolic acid, even in the largely diluted form in which they used it, killed or injured so many of their sheep. And it may be well, just here, to remember, that no microbe-fearing surgeon, of any age, clime or country, has achieved greater success than the great gynecological surgeon of Manchester, the plain tap-water Lawson Tait, who, in his long and large surgical practice, has never used *any* antiseptics, in even the foulest cases.

3d. As to tamponing the uterine cavity and vagina, after they are emptied, and supposed to be surgically clean, it is a measure we fail to see what good purpose it should serve; whilst such tampons, must, unavoidably prove a source of painful irritation, and serious disturbance to the patient.

4th. The proposition that a physician should be held criminally liable for *manslaughter*, if an aborting patient should die while under his care, simply because said physician had failed to treat said patient by the application of carbolic acid to the endometrium and vagina, and medicated uterine tampons—or by any other *specified* means or remedies—is plainly absurd, intensely pernicious, practically dogmatic, and would be dangerous, were its source authoritative. Even the *spirit* of such a proposition, strongly smacks of the horrible times of inquisitorial intolerance, and startlingly calls forth visions of torturing rack, worthy of the narrow bigotry of the "Holy Church," during the bloody, cruel hideous night of the dark ages, when mankind were slaves, and liberty and intellectual enlightenment had fled the earth.

Mr. President, just for a moment, let us consider the worse than savage cruelty of this abominally fanatical proposition: That simply because good old Dr. Pray had not freely applied carbolic acid to all his aborting patients, and one of them should die, that therefor, this true, noble and ever faithful self-sacrificing old servant of the people, should be straightway ignominiously stript of his well earned honors and citizenship, forced to don felon's stripes, and by brutal minions, harshly driven away to wear out the remnant of his declining days in public disgrace and sorrow, is such a henious crime, that free born Americans will never permit, the iron-clad fanatical dogmas from the land of the "Lord's annointed" to the contrary, notwithstanding.

